# On the Surface Residual Stress Measurement in Magnesium Alloys Using X-Ray Diffraction

**DOI:** 10.3390/ma13225190

**Published:** 2020-11-17

**Authors:** Amir Yazdanmehr, Hamid Jahed

**Affiliations:** Fatigue & Stress Analysis Laboratory, Mechanical & Mechatronics Engineering Department, University of Waterloo, 200 University Ave. W., Waterloo, ON N2L 3G1, Canada; hamid.jahed@uwaterloo.ca

**Keywords:** residual stress measurement, X-ray diffraction, magnesium alloys, stress correction factors, shot peening

## Abstract

X-ray penetration in magnesium alloys is significant due to the low X-ray mass attenuation coefficient. To measure the surface residual stresses in magnesium alloys, a correction needs to be made to account for penetration depth. The residual stresses in as-received and shot peened AZ31B-H24 rolled sheet samples were measured using two-dimensional X-ray diffraction (2D-XRD) method. The electro-polishing layer removal method was used to find the residual stress pattern at the surface and through the depth. The results show that the corrected residual stresses in a few tens of micrometers layer from the surface differ from the raw stresses. To better estimate the residual stress distribution in the surface, the grazing-incidence X-ray diffraction (GIXD) technique was applied. Additionally, micrographs of the lateral cross-section of the peened specimens confirmed the presence of microcracks in this region, causing the residual stresses to vanish. Due to the low X-ray absorption coefficient of Mg alloys, this study shows how a small uncertainty in a single raw measurement leads to high uncertainty in the corrected residual stresses. The results were corroborated with the hole drilling method of residual stress measurements. The corrected X-ray diffraction (XRD) results are in close agreement with the hole drilling and GIXD results.

## 1. Introduction

Magnesium (Mg), the lightest commercially available industrial metal, is of growing interest to automotive and aerospace industries which are seeking to increase fuel efficiency by decreasing vehicle weight. To expand the use of these alloys, among other studies, the role of residual stresses induced by different common manufacturing processes, such as machining and welding, or surface treatment processes, such as shot peening, laser shock peening and cold spray, needs to be studied and quantified.

X-ray diffraction (XRD) is a widely used non-destructive method for residual stress measurement. The sin^2^ψ method is the most common XRD method for residual stress measurement, and standardized approaches using this method are well-established [1]. The XRD method has been used to measure residual stress in Mg alloys in several studies. These studies use the sin^2^ψ method for stress measurement and layer removal to evaluate residual stress distribution through the depth. For the residual stress induced by shot peening process, Zhang and Lindemann [2], Liu et al. [3], Liu et al. [4], Zinn and Scholtes [5], and Bagherifard et al. [6] evaluated the residual stress distribution due to the shot peening of AZ80, Mg–10Gd–3Y, ZK60, AZ31, and AZ31B, respectively. All reported a compressive residual stress in the sub-surface layer of the shot peened samples. Other studies measured the residual stresses from welding Mg alloys at different distances from the weld centerline: friction stir welding of AZ31 [7], friction stir welding of ZK60 [8], tubular laser welding of AZ31 [9], butt joint welding of AZ31B and 304L steel alloy by hybrid laser-TIG [10], and laser beam welding of AZ31B [11]. The residual stress in the longitudinal and transverse directions of AZ91 welded by a CO_2_ laser as well as the in-depth distribution of residual stress using the layer removal method were studied by Kouadri and Barrallier [12]. Other researchers have measured the residual stresses induced in manufacturing various materials due to machining of AZ31B [13], due to dry and cryogenic machining of AZ31B on the surface and sub-surface in circumferential and axial directions [14], due to equal-channel angular pressing of AZ31 [15], and due to the extrusion of AZ31B [16]. A few studies have considered surface treatment processes such as cold spray [17,18,19] and laser shock peening [20].

Two stress correction factors are involved in measuring the residual stresses through the depth of material. The first regards the X-ray penetration depth and the second, for stress redistribution after layer removal [1]. The X-ray mass attenuation coefficient is a material constant that indicates the level of X-ray penetration through the depth of a material. The higher the coefficient, the lower the penetration through the depth. As Mg has a low density and low atomic number, its X-ray mass attenuation coefficient is very low, resulting in deeper X-ray penetration compared to materials with higher atomic numbers such as steel. In this case, measured surface residual stress by XRD will only be an average over a considerable depth and will require postprocessing to find real surface values.

Most of the mentioned literature on the measurement of the residual stress of Mg alloys have not discussed how penetration depth is handled when processing the XRD raw data. To this end, Table 1 summarizes the residual stress measurement studies on Mg alloys using XRD, showing the alloy, process, the measurement type, X-ray source, and whether the X-ray penetration and/or layer removal corrections are applied. Table 1 clearly shows, with some exceptions (e.g., Bagherifard et al., 2018 [6] and Shayegan et al., 2014 [18]), that applying stress correction factors has not been discussed in these studies. Further, a discussion of the experimental uncertainty of the corrected values based on the uncertainties in the observed stresses is missing in all of them.

In this study, we address the need to apply the depth of penetration correction factors to measure the surface residual stresses of Mg alloys. Here, we measure the residual stress induced by shot peening a sheet of AZ31B-H24. The stress correction factors from the X-ray depth penetration and layer removal are applied to raw data to evaluate the residual stress at each depth. Errors associated with the corrected stresses are calculated by combining the uncertainties in observed residual stresses and measurements of removed layers. This study also shows how measurement uncertainties are higher when the X-rays penetrate deeper into a material. The corrected stress profiles are compared with the two other independent measurements using the hole drilling method and grazing-incidence X-ray diffraction (GIXD) in the surface layer. To further verify the results obtained by the proposed correction factor, the surface topography of the lateral cross-section of the as-received and peened samples are evaluated using an optical microscope to study the effects of shot peening local damage on surface residual stresses.

## 2. Material, Experiments and Methods

### 2.1. Material

The material used in this study is a AZ31B-H24 rolled sheet with a 4-mm thickness (Luxfer MEL Technologies, Manchester, UK). The chemical composition of this alloy is shown in Table 2 [21]. The density, Young’s modulus of elasticity, and Poisson’s ratio are 1770 Kg/m^3^, 45 GPa, and 0.29, respectively [22].

### 2.2. Texture Measurement

Texture measurements were carried out on a Bruker D8 Discover X-ray diffractometer (Bruker AXS, Madison, WI, USA) equipped with a VÅNTEC-500 2D detector using Cu-$K_{\alpha }$ beam radiation at 40 kV and 40 mA, by measuring the incomplete pole figures of (0001), ($10\bar{1}0$), ($10\bar{1}1$), and ($1\bar{1}02$) planes for tilt angle ψ between 0° and 75° with a step of 15° and in axis rotation φ between 0° and 360° with a step size of 5°, as described in [23]. The complete pole figures were obtained using DIFFRAC.TEXTURE software, version 3.0.4, developed by Bruker AXS.

### 2.3. Shot Peening

Shot peening is widely used in the industry to enhance material fatigue strength. This process impinges small shots on the target surface at a velocity of 30–100 m/s. The impact of shots on the target surface results in plastic deformation of the surface layer, creating compressive residual stress near the surface of the target. Inducing the compressive residual stress is beneficial as it delays crack initiation and slows crack growth in the peened components. The Metal Improvement Company in Brampton, ON, Canada performed the shot peening of all samples. An Almen intensity of 0.4 mmN, a working distance of 10 cm, and vertical peening were used in all trials to create a full coverage on the Mg samples with the dimensions of 35 mm × 35 mm × 4 mm. Glass and steel shots with respective diameters of 350 and 280 μm were used. Roughness measurements were made using a Keyence VK-X250 confocal laser microscope (Keyence, Itasca, IL, USA) by scanning area dimensions of 1500 by 1000 μm. The roughness (Ra) values of samples before and after peening were 1.61 ± 0.03 and 4.88 ± 0.02 μm, respectively.

### 2.4. X-Ray Diffraction

**Figure 1:**
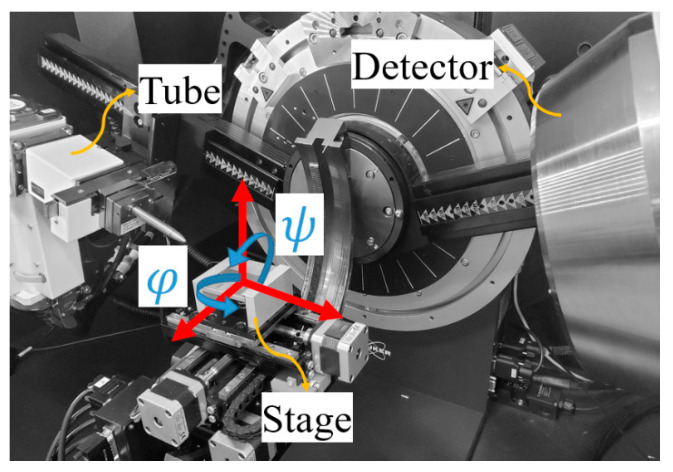
Sample orientation guide, defining different angles required for stress measurements with respect to the position of tube, sample and detector.

Residual stress measurements were performed on the samples using a Bruker D8-Discover equipped with a VÅNTEC-500 area detector with a radius of 135 mm. Cu-Kα radiation, as the most commonly used tube for XRD [24,25,26], was used at 40kV and 40 mA. The Cu source provides a better signal-to-noise ratio compared to the Cr-Kα when the substrate is a Mg alloy. The collimator size was 1.0 mm. Samples were mounted on a motorized stage and oscillated under amplitudes of 1.3 and 1.2 mm, at speeds of 3.5 and 5.5 mm s^−1^ for the X and Y axis, respectively. Oscillating the sample also has the benefit of measuring the residual stress based on the stress distribution at different locations. In this case, the results would be the average residual stress in the X-ray exposed area. The area detector captures a part of the Debye ring. In this paper, 20° from the Debye rings (γ = −80° to −100°) were used for stress measurements. This range was divided into 30 sub-regions, and the diffraction angle in each sub-region was calculated using the sliding gravity method. All 30 diffraction angles were involved in residual stress measurements. A sensitivity analysis was performed to confirm the evaluation method and parameters. According to Bragg’s law, the lattice spacing, d, is related to the diffraction angle, so any change in the d shifts the diffraction angle to the left or right. The diffraction angle (2θ) shifts can be used to calculate residual stress. In general, to obtain the stress tensor at a point, there are six independent unknown stresses. Thus, to evaluate the residual stress components, the sample should be tested in different orientations to find all the stress values. Different angles and orientations of the sample in XRD are illustrated schematically in Figure 1.

The orientations used for each residual stress measurement are listed in Table 3.

Based on the results of X-ray exposure time, samples were scanned for 60 s at each orientation. The Debye–Scherrer diffraction rings were collected using the area detector in a 2D diffraction image. The planes of $\left(11\bar{2}4\right)$ ($2\theta_{0}=99.22^{{}^{\circ}}$), $\left(20\bar{2}3\right)$ ($2\theta_{0}=90.45^{{}^{\circ}}$), and $\left(21\bar{3}1\right)$ ($2\theta_{0}=96.833^{{}^{\circ}}$) were used for stress measurements.

Equation (1) shows the system of equations that were solved to obtain the residual stress tensor. It shows the direct relationship between the stress components and diffraction angles by removing the strain components as intermediate parameters between diffraction angles and stress components.

(1) $$p_{11}\sigma_{11}+p_{22}\sigma_{22}+p_{33}\sigma_{33}+p_{12}\sigma_{12}+p_{13}\sigma_{13}+p_{23}\sigma_{23}=F\left(\theta_{0},\theta \right)$$

$p_{\mathrm{ij}}$ represents functions of sample orientations and also elastic constants of the material [28]. For stress measurement, first, strain components are calculated based on the diffraction angle change from $\theta_{0}$, diffraction angle in the stress-relieved sample, to θ, diffraction angle after applying stress, which in turn defines the changes in lattice spacing distance. Then, using Hook’s law, the strain components are converted to the stress based on the plane stress state of stress ($\sigma_{33}$ is assumed to be zero). Elastic constants (E, υ) of planes used in measurements are (43,995 MPa, 0.29 for $\left(11\bar{2}4\right)$), (43,995 MPa, 0.29 for $\left(20\bar{2}3\right)$), and (44,984 MPa, 0.29 for $\left(21\bar{3}1\right)$), respectively. All residual stress calculations were carried out by the Leptos software.

### 2.5. Layer Removal for Through-Depth Measurement

To measure the residual stress through the depth, the layer removal method using an electro-polishing device was employed. Electro-polishing, a chemical etching process, was used to remove a thin layer of surface material. In this process, a sample is made to act as the anode in an electrolytic cell. A thin material layer is removed without inducing any residual stress that can happen in mechanical layer removal. A probe is placed at the surface of the sample and by applying a voltage over a specific time, the material is removed via the electro-polishing process. For this purpose, the Proto electrolytic Model 8818-V3 (Proto Manufacturing Inc., Taylor, MI, USA), working at a voltage of 50V and with a probe diameter of 15 mm, was used to remove a layer without inducing residual stresses. The electrolyte was a mixture of ethanol 95%, distilled water, and perchloric acid 60%, based on the ASTM-E1558–09 [29]. To measure the residual stress through the depth, first, the stress was measured at the surface using XRD, then a few micrometers (approximately 20 μm) of the surface were removed by the electro-polisher, and the actual depth of polishing was measured using a dial indicator. The newly revealed surface was then exposed to the X-ray for stress measurement.

### 2.6. Stress Correction Methods

**Figure 2:**
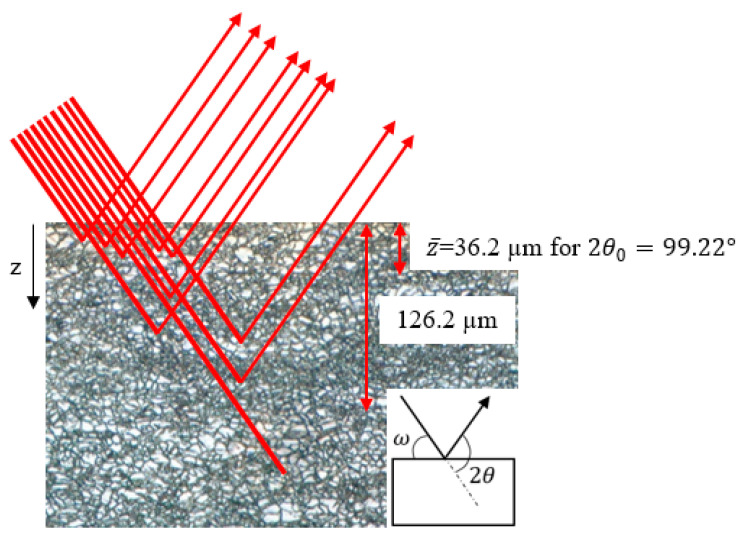
X-rays diffracting from different locations through the depth, 36.2 and 126.2 μm showing 50% and 90% ray diffraction, when using Cu-K_α_ for $2\theta =99.22^{{}^{\circ}}$ in Mg substrate.

There are two stress correction factors that were applied: first, for X-ray penetration depth and, second, for stress redistribution after layer removal [1]. The first stress correction factor, related to X-ray depth penetration, is necessary to measure the residual stress at the surface of Mg alloys because of their low mass attenuation coefficients (39.79 cm^2^/gr for the Cu-Kα beam) [30]. Respectively, 50% and 90% of the exposed X-rays were diffracted from the surface up to 36.2 μm and 126.2 μm through the depth. These penetration depths for steel alloys, in which the mass attenuation coefficient is 299.7 cm^2^/gr [30], are 1.1 and 3.7 μm for 50% and 90% X-ray diffraction, respectively. As such, for steel alloys, the observed stress can be considered as a surface (actual) stress, but in Mg alloys, the stress correction factor should be applied to compensate for the volume of element exposed to the ray. Consequently, by measuring the stresses before and after electro-polishing layer removal and knowing the depth of the removed layer, the actual residual stress at the surface can be evaluated. Figure 2 shows the concept of the diffraction of X-rays from different locations through the depth, suggesting that the observed residual stress would be the weighted average of residual stress at different locations through the depth. In this figure, ω and 2θ represent the incident angle and diffraction angle, respectively. This figure schematically shows that 50% and 90% of X-rays are diffracted up to 36.2 and 126.2 μm below the surface, respectively, and 10% of beams will penetrate deeper.

The method for correcting the observed residual stress after each electro-polishing step is described in [1] and [31]. The ratio, *G_Z_*, of the diffracted beam intensity from the surface to the depth z, *I_Z_*, to the total impinged intensity, *I_T_* is calculated using Equation (2),

(2) $$G_{z}=\frac{I_{z}}{I_{T}}=1-e^{-\mu z\left(\frac{1}{\sin\omega }+\frac{1}{\sin\left(2\theta -\omega \right)}\right)}$$

where μ, z, ω and 2θ are the linear absorption coefficient, depth, incident angle, and diffraction angle, respectively. The linear absorption coefficient is calculated by multiplying the density by the mass attenuation coefficient. By defining A as shown in Equation (3),

(3) $$A=\mu \left(\frac{1}{\sin\omega }+\frac{1}{\sin\left(2\theta -\omega \right)}\right)$$

Equation (2) can be rewritten as Equation (4).

(4) $$G_{z}=1-e^{-Az}$$

Owing to the low mass attenuation of magnesium and within the depth of X-ray penetration, the diffraction intensities from material points closer to the surface are higher than the deeper ones. An exponential weight function, in view of Equation (4), can be employed as a weighted average of stress to calculate the observed stress at each depth by Equation (5) [31].

(5) $$\hat{\sigma }\left(z\right)=\frac{\int_{z}^{\infty }\sigma \left(\tau \right)e^{-A\left(\tau -z\right)}d\tau }{\int_{z}^{\infty }e^{-A\left(\tau -z\right)}d\tau }=A\int_{z}^{\infty }\sigma \left(\tau \right)e^{-A\left(\tau -z\right)}d\tau$$

By differentiating Equation (5) with respect to *z*, the corrected stress (σ) can be evaluated from the measured (observed) residual stresses ($\hat{\sigma }$), using Equation (6) [1].

(6) $$\sigma_{i}={\hat{\sigma }}_{i}-\frac{1}{A}\left(\frac{{\hat{\sigma }}_{i+1}-{\hat{\sigma }}_{i}}{z_{i+1}-z_{i}}\right)$$

**Figure 3:**
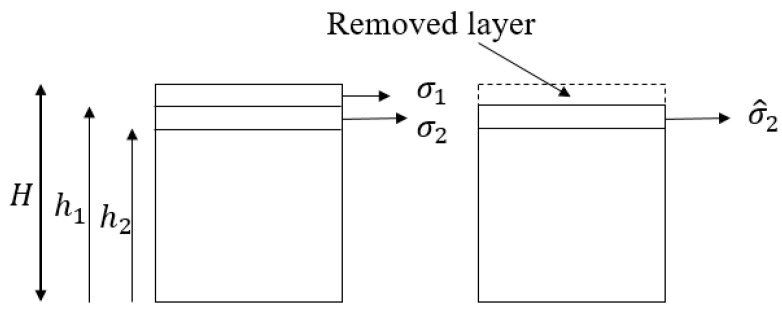
Stress correction factor due to redistribution of residual stress after layer removal and the parameters defined in Equation (7).

The second correction factor is concerned with the redistribution of residual stresses after removing a layer. When a stressed layer is removed, the residual stress measured in the sub-surface layer must be corrected to consider the effect of stress relaxation created by removing that stressed layer. Figure 3 illustrates the concept of the correction factor due to the redistribution of residual stresses after layer removal, showing the actual residual stresses $\sigma_{i}$ before polishing, plus the measured residual stresses after each layer removal step ${\hat{\sigma }}_{i}$.

Using the theory of elasticity and Taylor’s series, Equation (7) provides the method for stress correction after layer removal for a flat sample [1], where H is the initial thickness of the substrate and $h_{i}$ is the updated thickness of the material after layer removal.

(7) $$\begin{array}{cc} \sigma_{1}={\hat{\sigma }}_{1} & \mathrm{For}\;\mathrm{surface}\;\mathrm{point}\\ \sigma_{i}={\hat{\sigma }}_{i}-4{\hat{\sigma }}_{i-1}(\frac{h_{i}-h_{i-1}}{H-h_{i-1}}) & \mathrm{For}\;\mathrm{other}\;\mathrm{points}\;\mathrm{at}\;\mathrm{depth}\;z_{i} \end{array}$$

### 2.7. Grazing-Incidence X-Ray Diffraction Method (GIXD)

There are two methods to measure the residual stress distribution through the depth of materials using XRD: layer removal and grazing-incidence X-ray diffraction (GIXD). Layer removal is widely used for polished surfaces to remove a thin layer using an electro-polisher. GIXD is a non-destructive method that uses different low incident angles to provide different depths of penetration [1,28,32,33]. Depending on the material’s mass attenuation coefficient, the maximum penetration depth achievable with the GIXD method varies significantly. The GIXD method also requires the application of a corresponding stress correction factor [28,32,34].

As shown in Equation (2), the X-ray depth penetration is a function of the linear absorption coefficient μ, incident angle ω, and diffraction angle 2θ. Thus, to change the penetration depth, one or a combination of these three parameters should be modified. The first approach to change the X-ray penetration depth starts with a change in the linear absorption coefficient and uses incident rays with a different energy [35]. The second approach uses different diffraction angles and has been used to measure the residual stress in thin films [36] and in coatings [33]. The third approach uses various incident angles, which provide different penetration depths in each measurement [32,37,38]. The relation of the incident angle and X-ray depth penetration provides an opportunity to obtain the stress profile within a few to several tens of micrometers without layer removal in Mg alloys. In this method, a corresponding depth, $\bar{z},$ for an incident angle is defined as a thickness in which 50% of the impinged rays are diffracted up to the that depth [28]. So, considering $G_{z}=0.5$, the associated effective depth can be calculated by Equation (8).

(8) $$\bar{z}=\frac{0.693sin\omega .sin\left(2\theta -\omega \right)}{\mu \left(sin\omega +sin\left(2\theta -\omega \right)\right)}$$

Therefore, the corrected stress values are calculated, as shown in Equation (9) [28].

(9) $$\begin{array}{lll} \sigma_{1}={\hat{\sigma }}_{1} & & \mathrm{For}\;\mathrm{surface}\;\mathrm{point}\\ \sigma_{i}=\left({\hat{\sigma }}_{i}-{\hat{\sigma }}_{i-1}\right)e^{\left(\frac{{\bar{z}}_{i-1}}{{\bar{z}}_{i}}\right)}+{\hat{\sigma }}_{i-1} & & \mathrm{For}\;\mathrm{other}\;\mathrm{points}\;at\;depth\;{\bar{z}}_{i} \end{array}$$

To avoid the defocusing problem in small incident angles, a smaller collimator with a diameter of 0.3 mm was used in this measurement. Thus, the X-ray exposure time was increased to 12 min in each orientation to capture enough intensity. Table 4 shows the effective depth that X-rays penetrate Mg alloys using the Cu-Kα source, associated with each X-ray incident angle for $2\theta_{0}=99.22^{{}^{\circ}}$.

### 2.8. Error Calculation

This section provides a method for calculating errors in the corrected stresses due to layer removal and depth penetration corrections. The error of corrected stresses was calculated based on the root mean square approach. In this method, if R is a function of $X_{1}$, $X_{2}$, …, $X_{n}$ with respective errors of $\epsilon_{1}$, $\epsilon_{2}$, …, $\epsilon_{n}$ (Equation (10)),

(10) $$R=f\left(X_{1}\pm \epsilon_{1},X_{2}\pm \epsilon_{2},\ldots ,X_{n}\pm \epsilon_{n}\right)$$

then the error in R is calculated using Equation (11).

(11) ϵR=((ϵ1∂R∂X1)2+(ϵ2∂R∂X2)2+…+(ϵn∂R∂Xn)2)12

Similarly, for estimation of experimental uncertainties of corrected residual stresses, Equation (6) is rewritten as Equation (12).

(12) $$\sigma_{i}=f\left({\hat{\sigma }}_{i}\pm \epsilon_{{\hat{\sigma }}_{i}},\;{\hat{\sigma }}_{i+1}\pm \epsilon_{{\hat{\sigma }}_{i+1}},z_{i}\pm \epsilon_{z_{i}},z_{i+1}\pm \epsilon_{z_{i+1}}\right)$$

Then, the experimental uncertainties are calculated using Equation (13).

(13) ϵσi=((ϵσ^i∂σi∂σ^i)2+(ϵσ^i+1∂σi∂σ^i+1)2+(ϵzi∂σi∂zi)2+(ϵzi+1∂σi∂zi+1)2)12

Errors of the observed stress measurement before and after a polishing step, $\epsilon_{{\hat{\sigma }}_{i+1}}$ and $\epsilon_{{\hat{\sigma }}_{i}}$, were obtained to include the three measurements at different hkl planes, plus the error calculated by the Leptos software for each stress measurement. The accuracy of measuring the depths, $\epsilon_{z_{i+1}}$ and $\epsilon_{z_{i}}$, using the dial indicator was ±6 μm. The same approach was used for estimation of the experimental uncertainties after layer removal and GIXD method, shown in Equations (7) and (9), respectively.

### 2.9. Hole Drilling

Residual stresses were also measured using the hole drilling method. In this method, a strain gauge rosette is attached to the surface of a material and a drill tool creates a hole. By measuring the strain relaxation due to the drilling, the residual stress distribution through the depth can be calculated [39]. In this study, an MTS3000, Sint Technology hole drilling machine (Sint Technology, Calenzano, Italy) was used to measure the residual stress profile through the depth. Conventional HBM three-element strain gauge rosettes were installed on the surface of the AZ31B rolled sheet. The 400,000 rpm rotation speed of a drill tool with a 2-mm diameter was performed to create a shallow hole in the center of the strain gauge rosettes. The residual stresses were then evaluated using the non-uniform method [40], which has been used before for Mg alloys [17].

## 3. Results and Discussion

**Figure 4:**
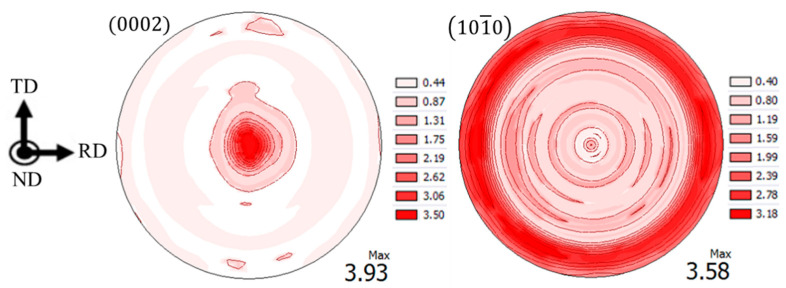
Texture of AZ31B-H24 rolled sheet used in this study.

Figure 4 shows the texture of the as-received material. It shows a strong basal texture in the normal direction (ND) of the sheet. This is a typical texture in wrought magnesium alloy, which is induced by cold/hot processes that reorient crystals along the process direction [23].

**Figure 5:**
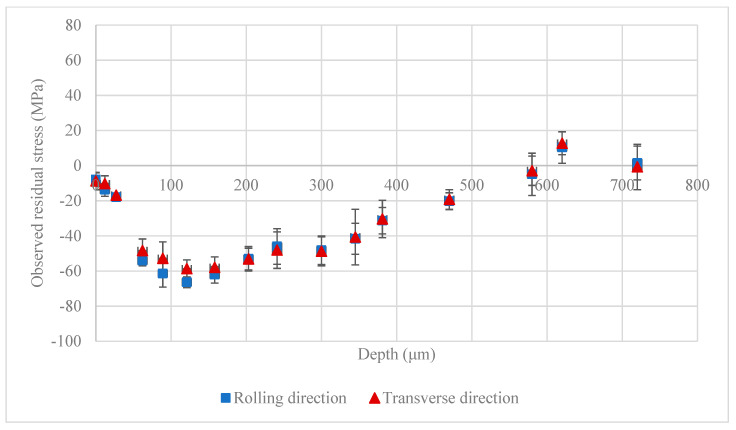
Observed (before proposed stress correction) residual stress on the peened AZ31B-H24 plate with steel shots; data points correspond to measurements after each layer removal.

Figure 5 shows the results of the observed residual stress (before stress correction) in the shot peened plate using the steel shot in the rolling direction (RD) and transverse direction (TD). The residual stress measurement results were obtained from the Leptos software, version 7.8, developed by Bruker AXS. The error bars in this figure were calculated based on three measurements at three lattice planes of $\left(11\bar{2}4\right)$ ($2\theta_{0}=99.22^{{}^{\circ}}$), $\left(20\bar{2}3\right)$ ($2\theta_{0}=90.45^{{}^{\circ}}$), and $\left(21\bar{3}1\right)$ ($2\theta_{0}=96.833^{{}^{\circ}}$). The figure shows that the residual stresses are very close to each other in the RD and TD, a fact which is in agreement with the axisymmetric nature of the texture along the ND (Figure 4). Although, as seen from Figure 4, the material has directional anisotropy, it has been shown that AZ31B sheet has similar behavior within the plane of the sheet in the RD and TD [22] which is in agreement with the residual stresses being similar in the RD and TD. From this point on, the reported residual stress measurement results are the average values of residual stresses in the RD and TD.

**Figure 6:**
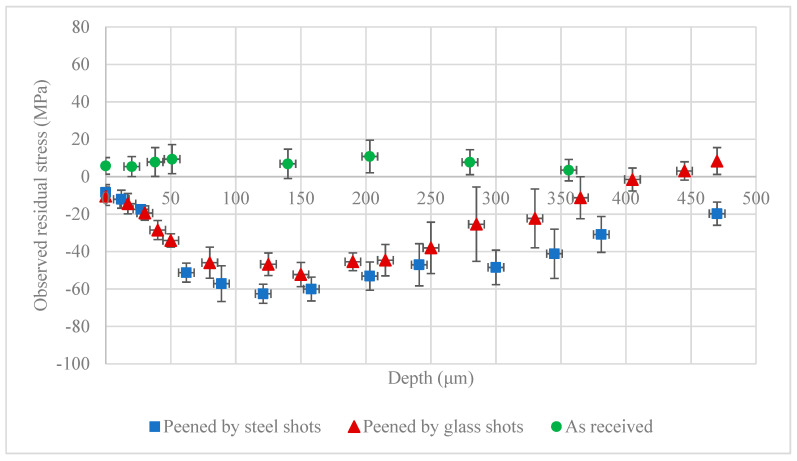
Observed (before proposed stress correction) residual stress on as received AZ31B-H24 plate and after shot peening with steel and glass shots.

Figure 6 shows the observed residual stress in the as-received sheet before the shot peening, and after shot peening with steel and glass shots. A small positive surface residual stress in the as-received sheet was observed. The as-received sample’s profile shows negligible residual stresses through depth, showing a uniform stress-relieved condition. The two residual stress profiles of the peened samples with steel and glass shots are similar, as the peening intensity in both cases is 0.4 mmN. The peened samples show compressive residual stresses at the surface and at the sub-surface layers, with the maximum compressive stresses at 100–150 μm below the surface. The two peened profiles are in good agreement in terms of the measurements of the surface residual stress, maximum compressive residual stress, and its corresponding depth. However, the steel shots show a slightly greater depth of compressive residual stress. These results are without consideration of the penetration depth and layer removal stress correction factors.

**Figure 7:**
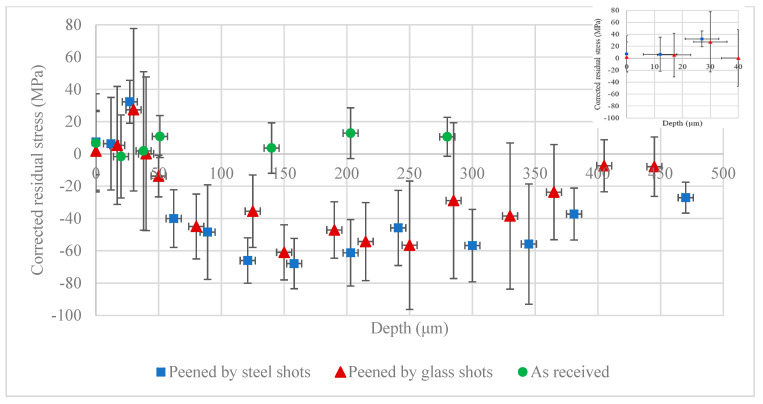
Corrected residual stress on the as-received AZ31B-H24 plate and after shot peening with steel and glass shots.

Using the method outlined in Section 2.6, the stress corrections for the depth of penetration and removal of layers were applied to the observed stresses, and the results are shown in Figure 7. This figure includes the results of corrected residual stress distributions before and after peening with steel and glass shots. The results show that the applied corrections change the residual stress values in the immediate vicinity of the surface but have minimal effect on the rest of the observed stresses away from the surface and through the depth of the sheet. While the observed surface residual stress profiles show compressive residual stresses, the average value of the corrected ones predict no residual stresses within the first 40 μm below the surface, as seen from the magnified inset figure of the first 40 μm below the surface in Figure 7. The corrected profile for the as-received sample shows a very similar stress distribution to its observed one where it shows negligible stresses through the depth, confirming an initial stress-relieved state.

The reason for the difference between the observed and corrected distributions near the surface in the peened sample is due to the low mass attenuation coefficient of Mg alloys and the presence of a stress gradient in the material. Because the surface measurement of residual stress in magnesium showed an average residual stress close to 120 μm, it may not be a true representation of the real surface residual stress. In the case where there is no stress gradient through the depth, akin to the initial residual stress state in the as-received case, the surface measurement is the real value of the residual stress, since the stress has the same value through the depth, including the first 120 μm. However, in the case where there is a stress gradient present, akin to the shot peen cases, the average residual stress resulting from surface measurement is not a true representation of the real surface stress. Successive measurements through the depth are required to correct the surface stress. Such successive measurements with the use of the method outlined in Section 2.6 allow for correcting the stress to account for the low mass attenuation coefficient of magnesium alloys. The correction in the case of shot peened samples studied here is significant, changing the average observed value in the first 40 μm from 40% of maximum residual stress to zero.

The observed stress distribution away from the surface, beyond 40 μm, does not show an appreciable difference from the corrected stresses. The difference between observed and corrected values at 100 μm is less than 5%, and the maximum values in both cases are around −60 MPa around 140 μm. The stress gradient in the vicinity of the surface in the case of shot peened samples is much larger than the one away from it. The stress value jumps from zero at the surface to −60 MPa in less than 100 μm, while it takes over 500 μm to return from the maximum value back to zero. In view of Equation (6), the difference between the successive observed stresses, ${\hat{\sigma }}_{i+1}-{\hat{\sigma }}_{i},$ close to the surface is more significant than away from it, resulting in less appreciable differences between observed and corrected stress away from the surface.

Comparing the error bars in Figure 6 and Figure 7, the results show that the measurement errors, and hence the uncertainty in measuring surface residual stress in Mg, grow considerably after correction. The errors increased from a maximum error of close to 50% from the average value in the observed stresses to over 150% in the case of corrected stresses. The error bars shown in Figure 6, uncorrected stresses, are the errors associated with experimental measurements as reported by Leptos software and are based on the resolution of the device measurement. The error bars shown in Figure 7 include the additional error/uncertainty due to corrections calculated from Equation (13). The low mass attenuation coefficient of Mg alloys, *A* in Equation (6), will result in larger error estimation. As parameter *A* is small for magnesium, an error in the estimation of $\left(\frac{{\hat{\sigma }}_{i+1}-{\hat{\sigma }}_{i}}{z_{i+1}-z_{i}}\right)$ is magnified by 1/*A*. Such an error is expected to be more significant in materials with small mass attenuation coefficients such as magnesium and less significant for materials with high mass attenuation coefficients such as steel alloys. The error in the depth measurements of layer removal method with a dial indicator is also considered in Equation (13), which in cases were up to 6 μm in around 20 μm of the removed layer. The relatively large uncertainty in the thickness of removed layer originates from two sources: the unevenness of the removed layer which is intrinsic in the employed chemical etching process, and resolution of the dial indicator. In view of Equation (7), $\left(\frac{h_{i}-h_{i-1}}{H-h_{i-1}}\right)$ will introduce additional error in estimation of the corrected stresses. This error can be reduced by using a laser-based profilometer instead of a dial indicator to reduce the uncertainty in the estimation of the polished depth.

**Figure 8:**
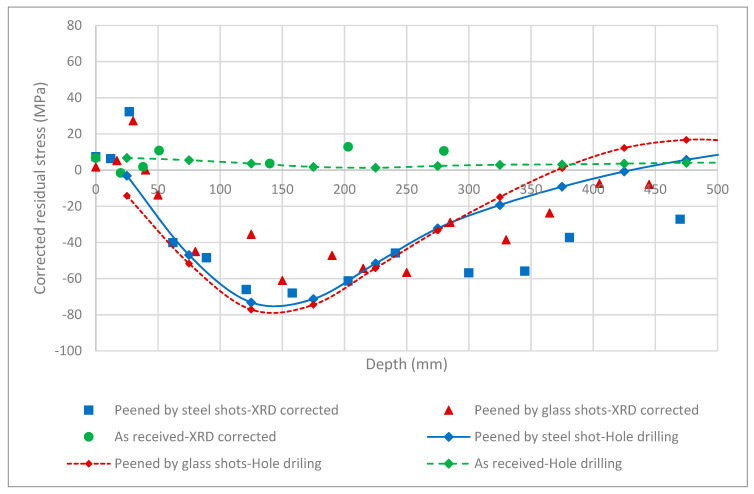
Residual stress distribution of the as-received, peened with steel shot, and peened with glass shot samples, as measured by the hole drilling method. Corrected residual stress measurements of XRD are added for comparison to show close agreement of the results.

To examine the results of corrected surface residual stress in as-received and shot peened samples, the hole drilling method, as an independent measurement method, was used to measure the residual stresses in the same samples. The results of the hole drilling measurements in as-received shots peened with steel shots and shots peened with glass shots are shown in Figure 8. A comparison of the hole drilling results with the corresponding corrected residual stresses from the XRD measurement is shown in the same figures. In all cases, the hole drilling results corroborate the corrected XRD results by showing similar residual stress at the surface and similar stress distribution through the depth. Albeit, the hole drilling method cannot provide a direct measure of the residual stress at the surface, because a hole needs to be drilled first to calculate the relaxed stresses. In the hole drilling tests, the first hole was drilled at the depth of 25 μm, as recommended by the standard. For both cases of shot peened samples, this first measurement corresponded to zero stress, corroborating the corrected residual stress measured by XRD. In the case of the as-received sample, the hole drilling measurements show a uniform state of the stress through the thickness, which agrees very well with XRD measurements, including the values close to the surface.

**Figure 9:**
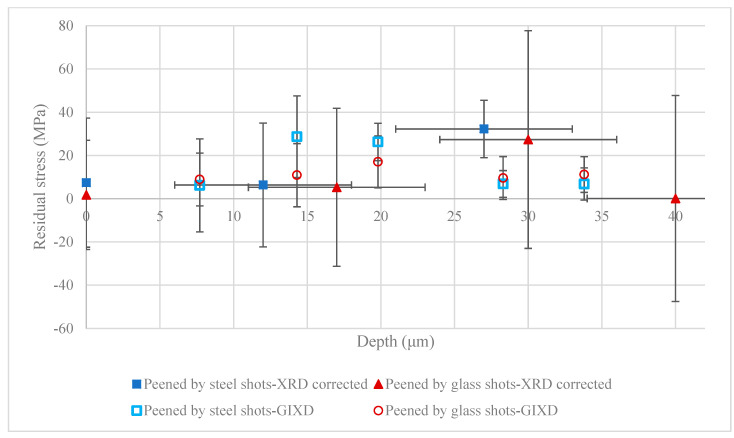
Results of the grazing-incidence X-ray diffraction (GIXD) method on the shot peened sample using steel and glass shots, and comparison with corrected stress evaluated by XRD.

To further examine the near surface XRD measurement results corrected by the proposed stress correction factors in the peened samples, the GIXD method was employed. The residual stresses in a thin layer from the surface were measured using GIXD. Figure 9 shows the results of the GIXD method on peened samples using steel and glass shots, and comparison with the corrected stresses evaluated by XRD. Again, the GIXD method’s results corroborate the corrected residual stresses, confirming that the stress correction factor’s impact on the residual stress measurements of Mg alloys. GIXD predicts negligible residual stresses up to 33 μm below the surface, similar to the predictions of the corrected residual stress profiles.

**Figure 10:**
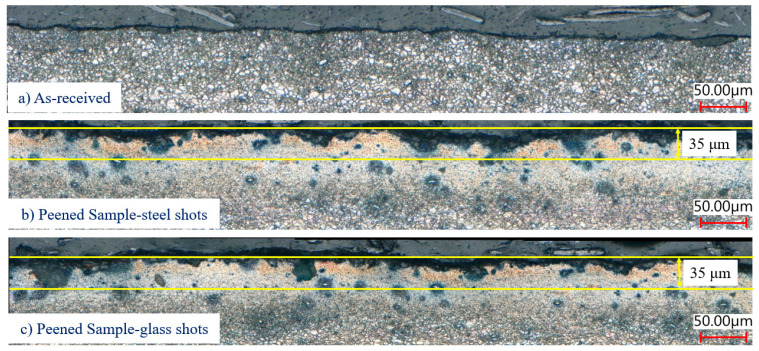
Cross-section of (**a**) the as-received sample, (**b**) peened using steel shots, and (**c**) peened using glass shots, showing much higher roughness in the shot peened samples as compared to the as-received sample.

To investigate the cause of these unexpected results, i.e., zero residual stress near the surface of the peened samples, the microstructures of the as-received and peened samples were examined. Samples from as-received and peened sheets near the surface were cut and polished, then observed under an optical microscope. Figure 10 shows the cross-section of the as-received and peened samples. It indicates that the as-received sample has a uniform and undamaged structure and smooth surface, but the peened samples show clear damage and much rougher surfaces, where the material was work hardened due to the peening. This figure also shows the presence of microcracks up to 35 μm below the surface, where the corrected residual stresses show negligible residual stress, even though the observed measurements show increasing compressive residual stress within this layer. In the presence of microcracks, the residual stresses are expected to vanish in this section. Thus, the corrected residual stress profiles agree with the surface topography of the peened samples. The reason for such surface deterioration could be associated with the hexagonal close-packed (HCP) structure of Mg, which does not have enough basal slip systems at room temperatures to accommodate uniform deformation [41,42,43], causing poor deformability at low temperatures. Another deformation mechanism in HCP metals is twinning [42,43]; however, only the extension twinning on the ($10\bar{1}2$) plane is active at room temperature [41,42,43]. Considering that shot peening created severe plastic deformation at the surface layer of a material, the limited deformability of magnesium alloys at room temperature significantly damages the surface.

## 4. Conclusions and Further Remarks

Several studies have used the XRD method for surface residual stress measurements; however, to date, the effect of the depth of X-ray penetration in Mg alloys on the results of measurements has not been adequately studied. To study this effect, the residual stress distributions of AZ31B-H24 before and after peening under two different peening conditions were measured using conventional two-dimensional X-ray diffraction (2D-XRD), GIXD, and hole drilling methods. For the evaluation of residual stress profiles through the depth, the electro-polishing method for layer removal was employed, and the critical effect of stress correction factors was discussed. It was shown that due to the low mass attenuation coefficient of Mg, which results in a high depth of penetration of Cu-Kα rays, the uncertainty of the surface residual stress measurement is high. To account for the high depth of penetration, a correction to the measured residual stress was proposed. The difference between the corrected and measured residual stress profiles was shown to be significant in the first 40 μm from the surface. Such a difference beyond the vicinity of the surface was not as significant. Considering that surface residual stresses are the main reason for delaying crack initiation and hindering crack growth near the surface, the proposed corrections for surface stresses in structural components made from Mg alloys is a necessary consideration. Therefore, conventional X-ray residual stress analysis in combination with the layer removal technique is a suitable tool to measure surface residual stresses and to detect stress gradients in the near surface region of magnesium alloys.

To verify the result of the stress corrections, two other methods were used: the GIXD technique and hole drilling. Both GIXD and hole drilling results were in good agreement with the corrected stresses, showing there is no compressive residual stress within a layer close to the surface for a shot peened magnesium sheet. Examination of as-received and peened cross-section near the surface revealed the surface damage within the 35 μm from the surface of the peened samples. It is believed that in this region stresses are released due to the local damage caused by the peening process. For the residual stress values beyond this layer, the observed and corrected residual stresses were close and in agreement with the hole drilling results.

The application of the proposed method into the design of structural parts made of magnesium with surface modification is crucial. The importance is twofold: (i) measured values from a single measurement at the surface is not representative of residual stress at the surface; (ii) the error associated with the surface residual stresses measured using the conventional X-ray method is much higher than one single measurement.

The first important aspect, (i), is the fact that fatigue cracks are generally initiated at the surface and such stresses in Mg cannot be measured unless the depth of X-ray penetration is considered. Using a conventional X-ray method, the proposed method should be used to obtain the real surface stresses. The presence of a beneficial (compressive) residual stress at the surface delays crack initiation leading to longer life. Crack initiation life is a crucial design criterion in many industries—e.g., automotive industries. The reason for surface modification technologies such as shot peening is to induce beneficial residual stress at the surface. Therefore, it is important to have the value of residual stress at the surface.

The second important aspect, (ii), is crucial in design reliability analysis. It was shown that error associated with the proposed correction for measurement of real residual stresses at the surface using a conventional X-ray method is considerably larger than the error associated with a single measurement. Such large errors must be considered in design reliability analysis of structural parts made of Mg with surface modification. This is particularly important if sub-surface residual stresses are required. This is an inevitable fact associated with the low mass attenuation property of Mg and use of conventional X-ray methods. Alternatively, and where only surface residual stresses are required, the GIXD method, which has significantly lower location error (error associated with x-axis in Figure 9) and relatively lower stress error (error associated with y-axis in Figure 9), may be employed.

## Figures and Tables

**Table 1:** Summary of studies on residual stress measurement of Mg alloys using X-ray diffraction (XRD).

Reference	Material	Process	Surface/In-Depth Measurement	X-Ray Source	Correction Applied
Zhang and Lindemann [2]	AZ80, Forged	Shot peening	In-depth	Cu-Kα	NA
Liu et al. [3]	Mg–10Gd–3Y, Extrusion	Shot peening	In-depth	NA	NA
Liu et al. [4]	ZK 60, Extrusion	Shot peening	In-depth	NA	NA
Zinn and Scholtes [5]	AZ31, Rolled	Shot peening	In depth	NA	NA
Bagherifard et al. [6]	AZ31, Rolled	Shot peening	In-depth	Cr-Kα	Yes
Commin et al. [7]	AZ31, Rolled	Friction stir welding	Surface	Cr-Kα	NA
Silva et al. [8]	ZK60, Cast	Friction stir welding	Surface	Co-Kα	NA
Nitschke-Pagel and Dilger [9]	AZ31B	Tubular laser welding	Surface	Cu-Kα	NA
Zeng et al. [10]	AZ31B, Rolled	Hybrid laser-TIG welding	Surface	NA	NA
Coelho et al. [11]	AZ31B, Rolled	Laser beam welding	Surface	NA	NA
Kouadri and Barrallier [12]	AZ91, Rolled	Laser beam welding	In-depth/Surface	Cr-Kα	NA
Outeiro et al. [13]	AZ31B, Rolled	Machining	In-depth	Cr-Kα	NA
Pu et al. [14]	AZ31B, Rolled	Machining	In-depth	Mn-Kα	NA
Hosaka et al. [15]	AZ31B, Extrusion	Equal-channel angular pressing	Surface	NA	NA
Kalatehmollaei et al. [16]	AZ31B, Extrusion	Machining	Surface	NA	NA
Marzbanrad et al. [17]	AZ31B, Rolled	Cold spray	Surface	Cu-Kα	NA
Shayegan et al. [18]	AZ31B, Extrusion	Cold spray	In-depth	Cr-Kα	Yes
Shaha et al. [19]	Mg alloy	Resistance spot welding	In-depth	NA	NA
Zhang et al. [20]	AZ31B, Rolled	Laser shock peening	In-depth	Cr-Kα	NA

**Table 2:** Chemical composition of AZ31B rolled sheet [21].

Composition	Al	Zn	Mn	Mg
Weight%	2.73	0.915	0.375	Bal.

**Table 3:** Different sample orientations [27].

*ψ* (°)	*φ* (°)
0	0
25	0, 45, 90, 135, 180, 225, 270, 315
50	0, 45, 90, 135, 180, 225, 270, 315

**Table 4:** Cu-Kα effective penetration depths for Mg alloys, corresponding to different incident angles, calculated from Equation (8).

*ω* (°)	*z* (μm)
5	7.7
10	14.3
15	19.8
25	28.3
35	33.8
